# Apolipoprotein CIII regulates lipoprotein-associated phospholipase A_2_ expression via the MAPK and NFκB pathways

**DOI:** 10.1242/bio.201410900

**Published:** 2015-04-02

**Authors:** Xiaolei Han, Tiedong Wang, Jifeng Zhang, Xingxing Liu, Zhuang Li, Gangqi Wang, Qi Song, Daxin Pang, Hongsheng Ouyang, Xiaochun Tang

**Affiliations:** 1 Jilin Provincial Key Laboratory of Animal Embryo Engineering, College of Animal Sciences, Jilin University, Changchun 130062, China; 2 Cardiovascular Center, Department of Internal Medicine, University of Michigan, Ann Arbor, MI 48109, USA

**Keywords:** Apolipoprotein CIII, Lipoprotein-associated phospholipase A_2_, MAPK pathway, NFκB pathway, Inflammation

## Abstract

Apolipoprotein CIII (apo CIII), a small glycoprotein that binds to the surfaces of certain lipoproteins, is associated with inflammatory and atherogenic responses in vascular cells. Lipoprotein-associated phospholipase A_2_ (Lp-PLA_2_) has been proposed as an inflammatory biomarker and potential therapeutic target for cardiovascular disease (CVD). Here, we report that apo CIII increases Lp-PLA_2_ mRNA and protein levels in dose- and time- dependent manner in human monocytic THP-1 cells, and the increase can be abolished by MAPK and NFκB pathway inhibitors. Lp-PLA_2_ inhibitor, 1-linoleoyl glycerol attenuates the inflammation induced by apo CIII. In turn, exogenous Lp-PLA_2_ expression upregulates apo CIII and the upregulation can be inhibited by 1-linoleoyl glycerol in HepG2 cells. Moreover, plasma Lp-PLA_2_ level is correlated with apo CIII expression in pig liver. *In vivo*, Lp-PLA_2_ expression in monocytes and its activity in serum were significantly increased in human apo CIII transgenic porcine models compared with wild-type pigs. Our results suggest that Lp-PLA_2_ and apo CIII expression level is correlated with each other *in vitro* and *in vivo*.

## Introduction

Atherosclerosis is a chronic inflammatory disease that is associated with hypertriglyceridemia, hypercholesterolemia and vascular cell dysfunction. Apolipoprotein CIII (apo CIII), a small glycoprotein, is synthesized by the liver and to a lesser extent by the intestines, is one of the major components of triglyceride-rich lipoproteins (TRL) (Aalto-Setälä et al., 1992). The overexpression of human apo CIII in mice, rabbits and pigs has been shown to reduce lipoprotein lipase (LPL) activity and lead to hypertriglyceridemia, which is associated with atherosclerosis (Ding et al., 2011; Ito et al., 1990; Wei et al., 2012). Numerous *in vitro* studies have demonstrated that apo CIII can promote proatherogenic responses in endothelial cells (ECs) and macrophages. For example, apo CIII increases vascular cell adhesion molecule-1 (VCAM-1) and intercellular cell adhesion molecule-1 (ICAM-1) expression in ECs, causing the adhesion of THP-1 cells to ECs. In addition, apo CIII-enriched apolipoprotein B lipoproteins enhance the adhesion of human monocytes to ECs (Caron and Staels, 2008; Kawakami et al., 2006a; Kawakami et al., 2006b).

Lipoprotein-associated phospholipase A_2_ (Lp-PLA_2_) is a calcium-independent, secreted phospholipase A_2_ that binds to circulating lipoproteins and catalyzes the hydrolysis of oxidized LDL with a truncated sn-2 acyl chain to release inflammatory products, oxidized fatty acids and lysophosphatidylcholine (LysoPC) (Rosenson and Stafforini, 2012; Stafforini et al., 1987; Tew et al., 1996; Zalewski and Macphee, 2005). These products have been shown to cause pro-inflammatory and pro-apoptotic effects *in vitro* studies (Matsumoto et al., 2007; Wang et al., 2010). In addition, increased Lp-PLA_2_ expression has been observed in necrotic cores and in macrophages of vulnerable and ruptured plaques from human and rabbit atherosclerotic lesions (Häkkinen et al., 1999; Lavi et al., 2007). The WOSCOPS (West of Scotland Coronary Prevention Study) was the first report that demonstrated the plasma concentration of Lp-PLA_2_ has a strong and positive association with the risk of coronary events, even when age, systolic blood pressure, and lipoprotein levels were put into consideration (Packard et al., 2000); these results were confirmed in subsequent investigations (Hatoum et al., 2011; Sabatine et al., 2007). The importance of Lp-PLA_2_ as an independent biomarker of CVD remains controversial (Mallat et al., 2010; Thompson et al., 2010) due to recent clinical investigations (White et al., 2014) that contradict the positive association reported by the WOSCOPS. However, Darapladib, an Lp-PLA_2_ inhibitor, decreased the levels of interleukin (IL)-6 and high-sensitivity C-reactive protein (hs-CRP) by 12.3% and 13%, respectively, after the oral administration of 160 mg daily for 12 weeks (Mohler et al., 2008) and significantly halted the necrotic core volume increase compared to a placebo at 12 months (Serruys et al., 2008). Therefore, investigation of the factors that regulate Lp-PLA_2_ levels is needed.

Here, we investigated the effects of apo CIII on Lp-PLA_2_ expression. In addition, we previously developed a genetically modified, human apo CIII overexpression porcine model, which has a longer triglyceride absorbance and clearance time than the wild types and exhibits a 2.5-fold and 2.3-fold increase in plasma triglycerides in the postprandial and fasting states, respectively (Wei et al., 2012). In these models, plasma Lp-PLA_2_ activity and expression are also investigated. These data may benefit to understanding the regulation of Lp-PLA_2_ and the relationship with apo CIII *in vitro* and *in vivo*.

## RESULTS AND DISCUSSION

### Effects of apo CIII on Lp-PLA_2_ gene expression

**Fig. 1.**
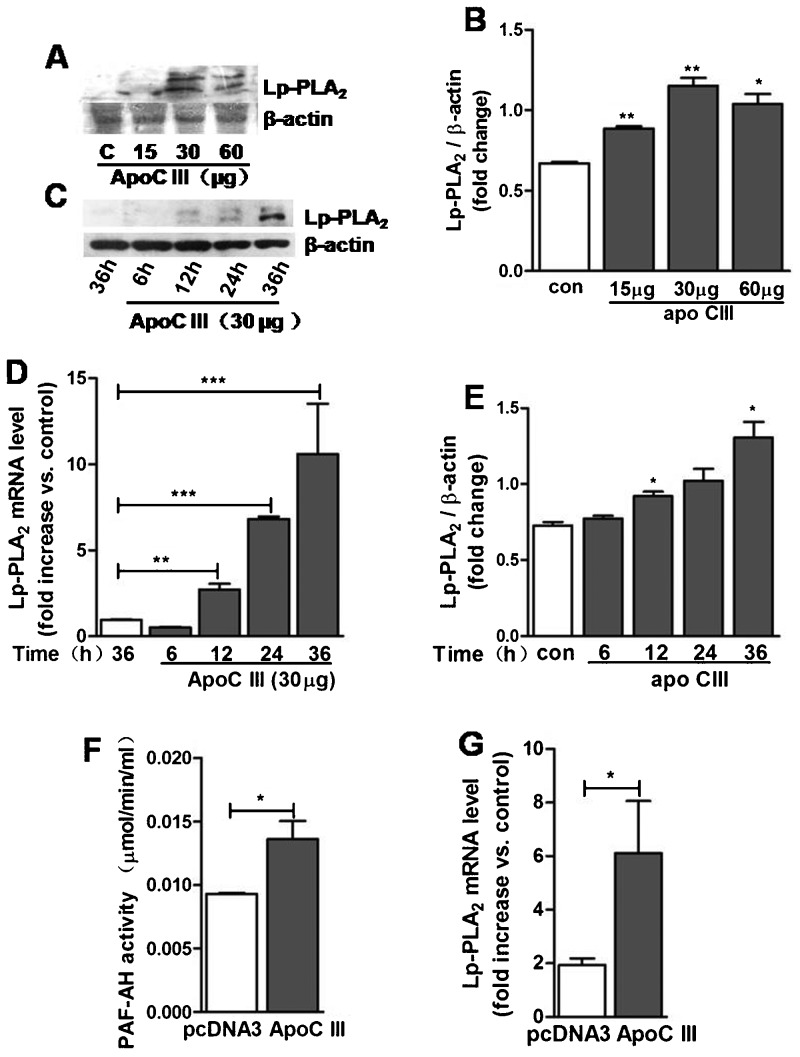
Effects of apo CIII on Lp-PLA_2_ expression. (A,B) THP-1 cells were incubated with the indicated concentrations of apo CIII for 48 h. Lp-PLA_2_ expression was tested by western blotting (*n* = 3). (C,D) THP-1 cells were incubated with 30 µg apo CIII for the indicated durations. The proteins were then collected, and Lp-PLA_2_ expression was examined by western blotting. (E) THP-1 cells were incubated with 30 µg apo CIII for the indicated durations. Lp-PLA_2_ mRNA was subsequently extracted and detected using quantitative PCR (*n* = 3). (F,G) THP-1 cells were transfected with a human apo CIII vector for 48 h (*n* = 3). The Lp-PLA_2_ activity was assayed using the PAF-AH assay kit, and the Lp-PLA_2_ mRNA level was determined using quantitative PCR. *P<0.05, **P<0.01, ***P<0.0001.

Lp-PLA_2_, as an independent biomarker and regulator of atherosclerosis, the level may be regulated by lipid associated factors. Apo CIII level is closely related with hypertriglyceridemia. To investigate the effect of apo CIII on Lp-PLA_2_ expression level, we treated human monocytic THP-1 cells with apo CIII. As shown in Fig. 1, the levels of Lp-PLA_2_ mRNA and protein expression were increased in THP-1 cells incubated with apo CIII in serum-free medium in a dose- and time-dependent manner (Fig. 1A–E). Furthermore, apo CIII-transfected THP-1 cells also exhibited increased Lp-PLA_2_ expression and activity (Fig. 1F,G). These data suggest that apo CIII can induce Lp-PLA_2_ expression *in vitro*.

### Lp-PLA_2_ involves apo CIII-induced inflammation

**Fig. 2.**
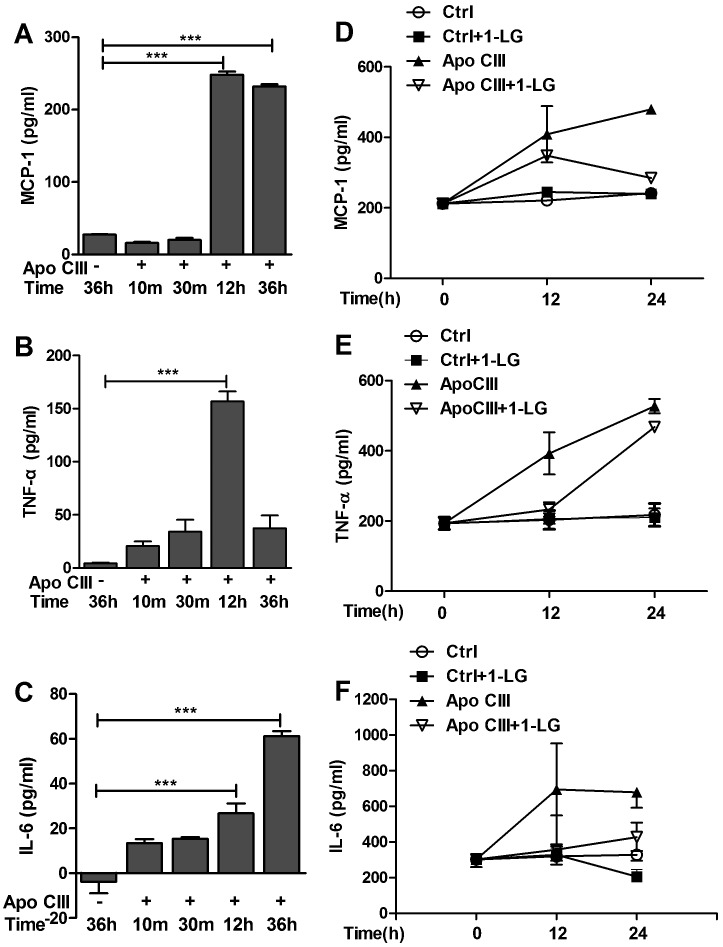
Lp-PLA_2_ involves in apo CIII-induced inflammation. (A–C) THP-1 cells were incubated with 30 µg of apo CIII for the indicated durations. MCP-1, TNF-α and IL-6 were detected in the medium using an ELISA kit (*n* = 3). (D–F) THP-1 cells were pretreated with 1-linoleoyl glycerol (75 µM) for 1 h and incubated with or without apo CIII (30 µg) for the indicated durations. The secretion of MCP-1, TNF-α and IL-6 was assayed using an ELISA kit (*n* = 3). Error bars represent mean±s.e.m. ***P<0.0001.

Apo CIII treatment significantly increases TNF-α, IL-6 and MCP-1 release from monocytic THP-1 cells (Fig. 2A–C). In circulation, Lp-PLA_2_ can act on oxidized LDL and produce oxidized fatty acids and lysophosphatidylcholine (LysoPC) which are proinflammatory factors. To address the question that if Lp-PLA_2_ mediates apo CIII's proinflammatory effect, the Lp-PLA_2_ inhibitor 1-linoleoyl glycerol was used to pretreat the cells before apo CIII treatment. As shown in Fig. 2D–F, the pro-inflammatory effects of apo CIII were attenuated by 1-linoleoyl glycerol, and no inhibitory effect was observed in the cells that lack apo CIII. These results suggest that Lp-PLA_2_ at least partially mediates the effects of apo CIII on TNF-α, IL-6 and MCP-1 release in monocytic THP-1 cells.

### Apo CIII stimulates Lp-PLA_2_ expression is regulated by MAPK and NFκB pathways

**Fig. 3.**
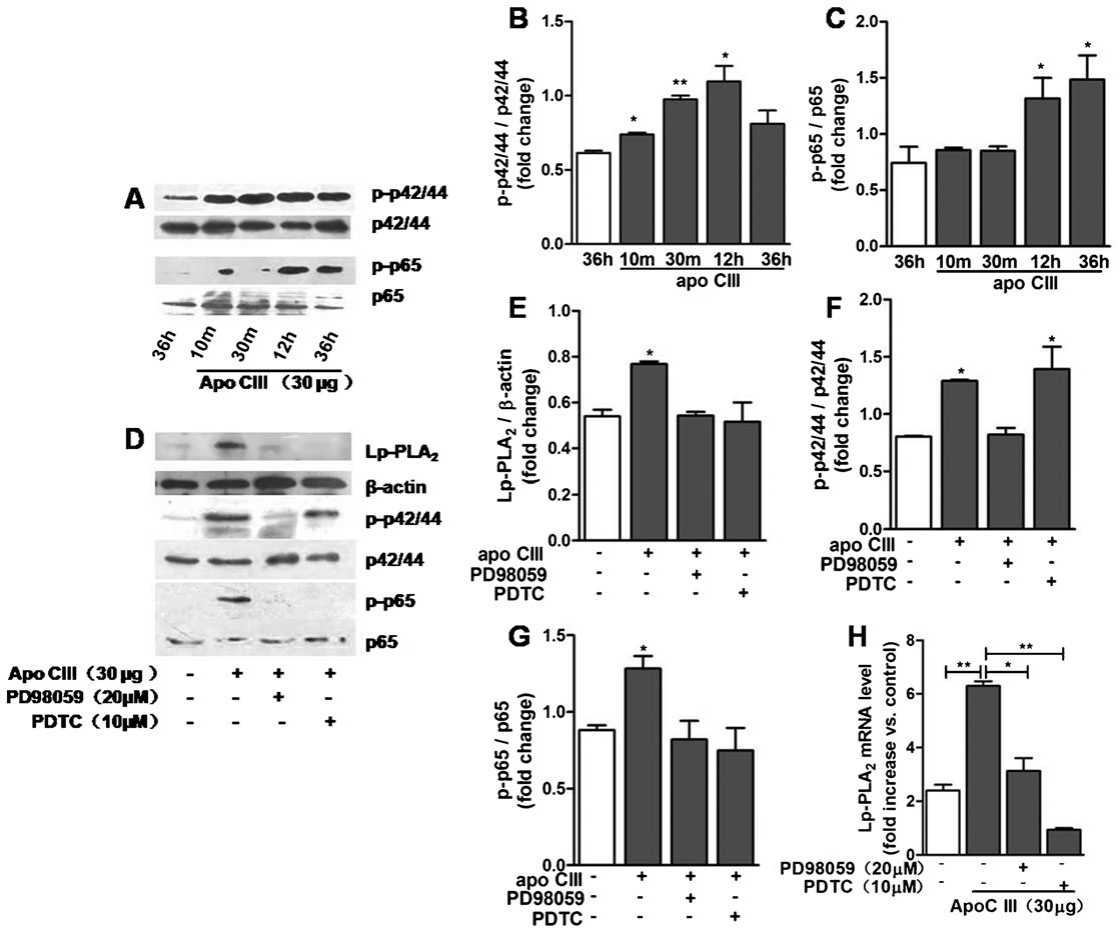
Apo CIII stimulated Lp-PLA_2_ increasing is regulated by MAPK and NFκB pathways. THP-1 cells were incubated with apo CIII and the indicated inhibitors. (A–C) After incubating with apo CIII for the indicated durations, THP-1 cell proteins were collected to detect p42/44 and p65 expression and phosphorylation using western blotting (*n* = 3). (D–G) THP-1 cells were incubated in PD98059 and PDTC for 1 h and then incubated with apo CIII, PD98059 and PDTC for an additional 12 h. The levels of Lp-PLA_2_, p42/44, p65 and their phosphorylated counterparts were determined by western blotting (*n* = 3). (H) THP-1 cells were treated as described in D, except that the incubation time was extended to 36 h. Quantitative PCR was performed to determine the levels of Lp-PLA_2_ mRNA expression (*n* = 3). Error bars represent mean±s.e.m. *P<0.05, **P<0.01.

The nuclear factor (NF)-κ B and mitogen-activated protein kinase (MAPK)-dependent pathways were closely related to the inflammatory response in cells. Other researcher and our previous report demonstrated that NF-κ B and MAPK pathway mediate the regulation of Lp-PLA_2_ gene expression. Therefore, here we assessed the possibility that both pathways involve in regulating Lp-PLA_2_ expression by apo CIII in THP-1 cells. Apo CIII stimulated the phosphorylation of p65 NFκB and p42/44 MAPK and upregulated Lp-PLA_2_ expression (Fig. 3A–D). The MAPK kinase inhibitor PD98059 and NFκB inhibitor PDTC can completely block the increase in Lp-PLA_2_ mRNA and protein expression induced by apo CIII (Fig. 3D–G). These results indicate that the p65 NFκB and p42/44 MAPK pathways mediate the upregulation of Lp-PLA_2_ expression induced by apo CIII.

### The relationship between apo CIII and Lp-PLA_2_ expression *in vivo* and Lp-PLA_2_ expression and activity in Apo CIII transgenic pigs

**Fig. 4.**
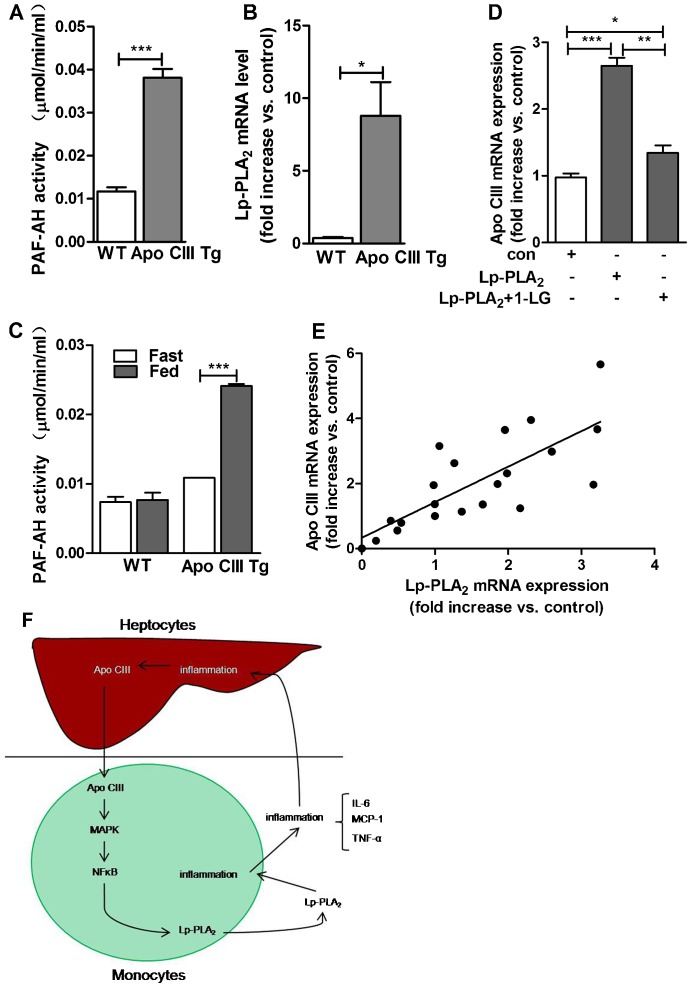
The relationship between apo CIII and Lp-PLA_2_ expression *in vivo* and Lp-PLA_2_ expression and activity in Apo CIII transgenic pigs. Blood samples from apo CIII transgenic and wild-type pigs were collected, and the plasma was isolated for use in Lp-PLA_2_ activity assays. Mononuclear cells were isolated for total RNA extraction. (A) The plasma Lp-PLA_2_ activity in the wild-type and apo CIII transgenic pigs was assayed less than 2 h after plasma isolation. (B) Total RNA was extracted from the mononuclear cells, and quantitative PCR for Lp-PLA_2_ mRNA level was conducted. (C) The wild-type and transgenic pigs were fasted for 16 h and fed olive oil for 2 h, the plasma was isolated, and the Lp-PLA_2_ activity were measured. *n* = 8 in the wild-type pigs, and *n* = 4 in the apo CIII transgenic pigs. Error bars represent mean±s.e.m. (D) Lp-PLA_2_ stable expressed HepG2 cells was incubated with 1-linoleoyl glycerol for 12 h. Quantitative PCR was performed to determine the levels of apo CIII mRNA expression (*n* = 3). (E) Blood and liver tissue were collected from slaughtered landraces, and quantitative PCR was performed to determine the levels of apo CIII mRNA expression in liver tissue and Lp-PLA_2_ mRNA expression in monocytes isolated from blood. Linear regression analysis is shown. *P<0.05, **P<0.01, ***P<0.0001. (F) A schematic model of apolipoprotein CIII regulates Lp-PLA_2_ expression.

Twenty pigs samples of blood and liver tissues were collected to observe the expression of apo CIII and Lp-PLA_2_, the results show that the mRNA level of apo CIII and Lp-PLA_2_ are correlated (Fig. 4E). The previously developed apo CIII transgenic pigs specifically overexpress human apo CIII in the liver and intestines (Wei et al., 2012). The model was used to confirm the correlation of apo CIII and Lp-PLA_2_ expression. When fed a normal chow diet, the apo CIII transgenic pigs exhibited a 4-fold increase in plasma Lp-PLA_2_ activity and a 10-fold increase of Lp-PLA_2_ mRNA in macrophage compared to the wild-type controls (Fig. 4A,B). There was no change in the plasma Lp-PLA_2_ activity in wild-type pigs in the fasting or fed (olive oil) states (Fig. 4C). Interestingly, the Lp-PLA_2_ activity can be increased in the apo CIII transgenic pigs after ingesting olive oil for 2 h (Fig. 4C). These findings demonstrate that apo CIII may be responsible for the changes in Lp-PLA_2_ expression at the transcriptional level.

In addition, we observed that exogenous Lp-PLA_2_ stable expressed in HepG2 cells could increase apo CIII mRNA level and the increased effects could be inhibited by 1-linoleoyl glycerol (Fig. 4D). Together these results suggested that increased apo CIII in liver could upregulate Lp-PLA_2_ expression via p65 NFκB and p42/44 MAPK pathways in macrophage in circulation. The increased Lp-PLA_2_ could stimulate macrophage inflammation independent with lipoproteins and the inflammatory factors in turn stimulate apo CIII expression in liver (Fig. 4F). The detailed mechanisms in the circle need to be further investigated.

## MATERIALS AND METHODS

### Animals and cells

The apo CIII transgenic pigs were developed using Chinese experimental miniature pig fibroblasts according to a previously described method, and they were fed a chow diet (Wei et al., 2012). For the experiments, wild-type and apo CIII transgenic pigs were administered olive oil orally (fat 23%) for fat load at 10 ml/kg body weight after a 16-h fast, and blood was collected for analysis. Human monocytic THP-1 cells were donated by Dr. Yang's lab (Jilin University) and maintained in RPMI-1640 medium with 10% fetal bovine serum (PAA, Austria). Pig monocytes were isolated from apo CIII transgenic or wild-type pigs using Histopaque-1077 (Sigma-Aldrich, USA). All of the animal experiments were conducted according to Jilin University Animal Care and Use Committee protocol no. 2008-11.

### Reagents

Apolipoprotein C-III was purchased from Sigma-Aldrich. The antibodies p42/44, p-p42/44, p65 and p-p65 were purchased from Cell Signaling. The antibodies Lp-PLA2 and β-actin were obtained from Bioss. PD98059 and PDTC were purchased from Beyotime, and 1-linoleoyl glycerol was obtained from Cayman. Human MCP-1, TNF-α, and IL-6 Elisa Kits were obtained from Boster.

### Immunoblotting

THP-1 cells were incubated with different combinations of reagents. For the expression analysis, the cells were lysed in cell lysis buffer (Beyotime, China) containing 1 mM PMSF and protein phosphatase inhibitor (Applygen, China) for 30 min on ice and were then centrifuged. The protein concentrations were determined using the Enhanced BCA protein assay kit (Beyotime, China). Equal amounts of protein were used for 12% SDS-PAGE and then transferred to nitrocellulose membranes. Immunoblots were performed with the indicated primary antibodies and the corresponding secondary antibodies. The signal was detected using BeyoECL Plus (Beyotime, China).

### Lp-PLA2 activity

Blood was collected from pigs using anticoagulant EDTA tubes and centrifuged at 1000 ***g*** for 10 min. The plasma was analyzed using a PAF Acetylhydrolase kit (Cayman, USA) according to the manufacturer's instructions.

### Quantitative real-time PCR

Total RNAs from the pig monocytes, THP-1 cells, HepG2 cells and liver tissues were extracted using the TRNzolA+ reagent according to the manufacturer's instructions (Tiangen, China), and 1 µg of RNA was used for reverse transcription (Invitrogen, China). The obtained cDNA was used for real-time PCR using the BioEasy SYBR Green I kit (Bioer, China) in an iQ^TM^5 system (Bio-Rad, USA) according to the manufacturer's recommendations. The expression of β-actin mRNA was used as an internal control to normalize target gene expression. Pig Lp-PLA_2_ primers, forward: 5′-CACTGACCTGGCATCTTAC-3′, reverse: 5′-TACCTGCTCGTTGCGTAG-3′; Human Lp-PLA_2_ primers, forward: 5′-TAATGATCGCCTTGACACCCT-3′, reverse: 5′-TACAGCAGCAACTATAAACCC-3′. Human apo CIII primers, forward: 5′-GCCACCAAGACCGCCAAGGAT-3′, reverse: 5′-GCAGGACCCAAGGAGCTCGCA-3′. Pig apo CIII primers, forward: 5′-AACCAGCGTGAAGGAGTCCGAG-3′, reverse: 5′-GTGAACTTGCCCTTGAACGTGC-3′.

### Quantification of proinflammatory cytokines

The levels of proinflammatory cytokines which stimulated by apo CIII and released in the culture media were evaluated by Human IL-6 ELISA Kit, Human TNFα ELISA Kit and Human MCP-1 ELISA Kit (Boster Inc., China), following the manufacturer's instructions.

### Statistical analyses

The data are expressed as the mean±SEM and were analyzed using a two-tailed unpaired *t* test in the GraphPad Prism software. *P*<0.05 was considered to be statistically significant.
